# Primary non-Hodgkin's lymphoma of the bladder: case report and literature review

**DOI:** 10.11604/pamj.2013.15.136.1599

**Published:** 2013-08-15

**Authors:** Tarik Mahfoud, Rachid Tanz, Mohamed Mesmoudi, Mohamed Réda Khmamouche, Basma El Khannoussi, Mohamed Ichou, Hassan Errihani

**Affiliations:** 1 Department of Medical Oncology, Military Hospital Mohammed V, Rabat, Morocco; 2 Department of Medical Oncology, National Institute of Oncology, Rabat, Morocco; 3 Department of Pathology, National Institute of Oncology, Rabat, Morocco

**Keywords:** Primary lymphoma, Non-Hodgkin's lymphoma, Bladder, Chemotherapy

## Abstract

Primary non-Hodgkin's lymphoma (NHL) of the bladder is a very rare entity. The clinical, radiological and endoscopic signs are not specifics. The diagnosis is exclusively histological. Chemotherapy, radiotherapy and surgery are the different therapeutic options used either alone or in combination. We report a 57 years old patient treated with chemotherapy (6 cycles of R-CHOP) for primary NHL of the bladder with a complete response while discussing the different specificities of this disease.

## Introduction

Most bladder tumors are derived from the epithelium. Non epithelial tumors of the bladder are extremely rare; and primary non-Hodgkin's lymphoma (NHL) of the bladder is even more exceptional [1–3]. Its presentation is nonspecific and its diagnosis is often a histological surprise. The treatment is not well defined and the prognosis remains unknown. We report a case of a patient followed in our department for a primary NHL of the bladder while reviewing the appropriate literature.

## Patient and observation

**Figure 1:**
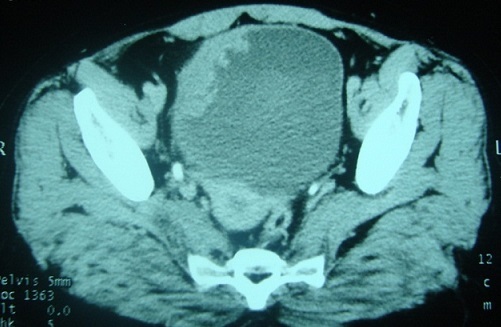
Tumor of the right anterolateral wall of the urinary bladder

**Figure 2:**
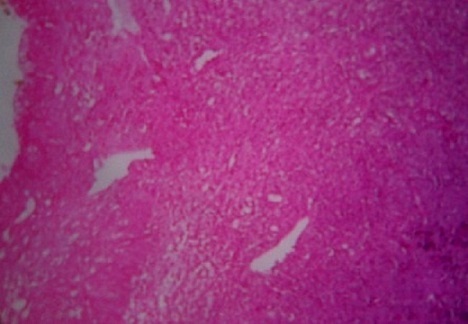
Aggregation of monomorphous population of large lymphoid tumor cells (hematoxylin-eosin, x 40)

**Figure 3:**
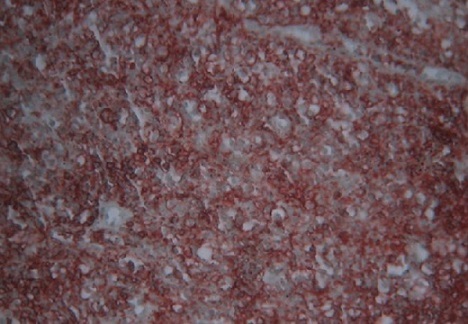
Immunohistochemical staining shows a positive reaction for CD20 (Avidine-biotine, x 40)

A 57-year-old woman with a history of recurrent cystitis which has been treated with repeated courses of antibiotics during a 5-year period. She has been experiencing 2 months of bladder irritation, dysuria, urinary urgency and terminal hematuria with presence of blood clots. Physical examination showed pelvic tenderness. Abdominal ultrasonography revealed a tumor of the right anterolateral wall of the urinary bladder that was confirmed by computing tomography (CT) scan (Figure 1). Cystoscopy showed an irregular, solid and ulcerative mass arising from the right wall of the bladder. Transurethral resection was performed and histology revealed a diffuse, dense infiltration of highly pleomorphic population of large cells (Figure 2). Tumors cells were strongly positive for CD79a, CD20 (Figure 3) and bcl2 protein. The proliferative fraction of cells, as determined by Ki-67 staining was 70%.

Immunohistochemical analyses were negative for cytokeratins and vimentine. This allowed us to retain the diagnosis of diffuse large B-cell lymphoma (DLBCL).

There was no cervical, thoracic, abdominal or pelvic lesion in the CT scan. Bone marrow biopsy showed no abnormality. A complete blood count and routine blood chemistry were normal. The disease was classified stage IE according to the Ann Arbor Staging system and the patient had a score of 0 by using the international prognostic index (IPI). Six cycles of R-CHOP regimen (Rituximab 375 mg/m^2^, cyclophosphamide 750 mg/m^2^, doxorubicine 50 mg/m^2^, vincristin 1.4 mg/m^2^ and prednisone 100 mg/day during 5 days; the protocol is repeated every 3 weeks) were given with complete response maintained after 48 months of follow up.

## Discussion

Secondary bladder involvement occurs in 10 to 20% of terminal NHL cases [1], while the primary NHL of the bladder are extremely rare and represent less than 1% of vesical tumors and less than 0.2% of extra-nodal lymphomas [2, 3]. This low prevalence is due to the poverty of the bladder in lymphoid tissue.

The etiology of primary NHL of the bladder has not been elucidated, because of the rarity of this pathology. According to some authors, lymphoma may be secondary to chronic cystitis [2, 4]. This could be the case of our patient who presented recurrent cystitis. However, this physiopathological hypothesis is contradicted by the fact that this history is present in only 20% of case with primary NHL of the bladder [5]. Other authors have raised the possibility of a residual embryonic cloaca, source lymphoid proliferation in adulthood [6].

Generally, primary NHL of the bladder affects six women for one man [1, 2]. Clinically, the disease is most often revealed by hematuria (80% of cases), but signs of bladder irritation (pollakuria, dysuria, urinary urgency) can be seen as in our case.

The location is most often retro-trigonal or lateral. The appearance on cystoscopy, ultrasound and CT scan is not different from that of urothelial tumors and it is histology combined with immunohistochemistry that affirms the diagnosis.

Histologically, it's usually a B-cell lymphoma. Indeed, small cell lymphocytic lymphoma and DLBCL are the most common; they each represent 30% of cases [7]. The BLBCL is characterized by a proliferation of atypical lymphoid cells. The tumor cells are of large size and often resemble normal centroblasts or immunoblasts. These cells express CD20, CD79a and bcl-2. The positivity of CD10, CD5 and Bcl6 remains variable. The differential diagnosis is essentially a poorly differentiated carcinoma; melanoma, burkitt's and Hodgkin's lymphoma; underlining the interest of an immunohistochemical panel in order to confirm the diagnosis. In our case, tumor cells did not express cytokeratins, vimentin but the expression of CD79a, CD20 and bcl2 protein was clearly positive, which enabled us to retain the diagnosis of lymphoma.

The staging should eliminate the presence of any other lesions to confirm the primary origin of the bladder lymphoma. It must include a total scan and a bone marrow biopsy.

Chemotherapy, radiotherapy and surgery, as well as combinations of these treatments are the therapeutics options for primary NHL of the bladder (Table 1). However, chemotherapy is the most often used in first intension and CHOP regimen is the most widely used protocol [1–3, 7]. The addition of rituximab in cases of CD20 positivity should be systematic due to the therapeutic benefit provided by this monoclonal antibody in the treatment of NHL. A second line chemotherapy or radiotherapy may be indicated in cases of locoregional recurrence. Radiotherapy can be used in first intention especially in low grades or in the adjuvant setting after resection but cannot constitute a standard treatment [8]. Indeed, its side effects are frequent and managing local recurrence after irradiation is very complicated. Concerning surgery; its place is limited; either in treatment of hydronephrosis by removing the obstruction and draining the urine that has accumulated upstream the obstruction or in some cases of localized low-grade tumors, transurethral resection may be performed followed by radiotherapy [7]. However, for locally advanced tumors or high grade, chemotherapy alone is recommended. That was the case of our patient who received six cycles of R-CHOP with a complete response maintained after 48 months of follow-up.

**Table 1:** Presentation, treatment and outcome of primary NHL of the bladder

Author	Age/Sex	Presentation	Histology	Treatment	Response	Follow up
Bates AW [1]	67/M	Hematuria and pain	DLBCL	CT + RT	Complete response	Alive after 16 years
Bates AW [1]	80/F	Hematuria and impaired renal function	DLBCL	RT	Complete response	Alive after 4 years
Bates AW [1]	84/F	Hematuria	DLBCL	None	Progression	Dead after 6 months
Antunes AA [2]	41/M	None	Follicular Lymphoma	CT	Not reported	Not reported
Leite KRM [3]	89/F	Urinary obstruction	DLBCL	CT	Complete response	Dead after 1 year
Al Maghrabi J [10]	69/F	Frequency and urgency	MALT	RT	Complete response	Alive after 5 years

M: Male/ F: Female/ DLBCL: Diffuse large B cell lymphoma; MALT: Mucosa Associated Lymphoid Tissue; CT: Chemotherapy; RT: Radiotherapy

The optimum follow-up protocol for patients treated for primary lymphoma of the bladder has yet to be determined. Follow-up evaluations should take place every 3 or 6 months for the first 2 years and yearly thereafter, as is the case for transitional cell carcinoma. It must include at least ultrasound examination and cystoscopy.

The prognosis is difficult to be determined given the rarity of primary NHL of the bladder. However, patients with high-grade lymphoma should be considered to have systemic disease and therefore, the IPI can be used to predict prognosis. In our case, the patient had an IPI score of 0; so it is part of the low risk with a 5-year survival estimated at 73% [9].

## Conclusion

Primary NHL of the bladder is a rare disease which diagnosis is exclusively histological. Chemotherapy should be used in first line especially in high grade or locally advanced tumor and it seems to give good results.

## References

[1] Bates AW, Norton AJ, Baithun SI (2000). Malignant lymphoma of the urinary bladder: a clinicopathological study of 11 cases. J Clin Pathol..

[2] Antunes AA, Nesrallah LJ, Srougi M (2004). Non-Hodgkin Lymphoma of the bladder. International Braz J Urol..

[3] Leite KRM, Bruschini H, Camara-Lopes LH (2004). Primary lymphoma of the bladder. International Braz J Urol..

[4] Acenero FMJ (1990). Primary malignant lymphoma of the bladder: Report of three cases. Pathol Res Pract..

[5] Simpson RH, Bridger JE, Anthony PP, James KA, Jury I (1990). Malignant lymphoma of the lower urinary tract: A clinicopathological study with review of the literature. Br J Urol..

[6] Aigen A (1986). Primary malignant lymphoma of urinary bladder. Urology..

[7] Peyromaure M, Van Glabeke E, Leblond V, Barrou B, Delcourt A, Richard F (2000). Le lymphome primitif de la vessie. Progrès en Urologie..

[8] Guthman DA, Malek RS, Chapman WR, Farrow GM (1990). Primary malignant lymphoma of the bladder. J Urol.

[9] Coiffier B (2005). State-of-the-art therapeutics: diffuse large B-cell lymphoma. J Clin Oncol..

[10] Al-Maghrabi J, Kamel-Reid S, Jewett M, Gospodarowicz M, Woodrow Wells, Banerjee D (2001). Primary Low-Grade B-Cell Lymphoma of Mucosa-Associated Lymphoid Tissue Type Arising in the Urinary Bladder Report of 4 Cases With Molecular Genetic Analysis. Arch Pathol Lab Med..

